# Exploratory Analysis of Practical Predictive Indices for the Efficacy of Mogamulizumab in Patients With Aggressive Adult T‐Cell Leukemia‐Lymphoma

**DOI:** 10.1002/hon.70114

**Published:** 2025-06-28

**Authors:** Yutaka Shimazu, Kenta Murotani, Hiroki Kitabayashi, Yukihiro Nishio

**Affiliations:** ^1^ Kyoto Innovation Center for Next Generation Clinical Trials and iPS Cell Therapy Kyoto University Hospital Kyoto Japan; ^2^ Department of Early Clinical Development Graduate School of Medicine Kyoto University Kyoto Japan; ^3^ Biostatistics Center Kurume University Fukuoka Japan; ^4^ Kyowa Kirin Co., Ltd. Tokyo Japan

**Keywords:** adult T‐cell leukemia‐lymphoma, LDH, LMR, mogamulizumab, predictive model

## Abstract

An exploratory analysis of past clinical trials was conducted to propose a predictive scoring system for the efficacy of mogamulizumab, an anti‐CC chemokine receptor 4 (CCR4) antibody, based on easily measurable parameters. Factors affecting progression‐free survival (PFS) were investigated using data from three clinical trials (NCT00920790, NCT01626664, and NCT01173887) and one clinical study (UMIN000013294) conducted in patients with relapsed/refractory (R/R) or untreated CCR4‐positive aggressive adult T‐cell leukemia‐lymphoma (ATL) receiving mogamulizumab treatment. Twelve routinely measured clinical parameters and three calculated indices—lymphocyte‐to‐neutrophil count ratio, platelet‐to‐lymphocyte count ratio, and lymphocyte‐to‐monocyte count ratio (LMR)—were selected as variables. Univariate Cox proportional hazards analysis identified albumin level, disease type, lactate dehydrogenase (LDH), monocyte count, neutrophil count, and LMR as relevant factors in R/R ATL patients treated with mogamulizumab monotherapy (*p* < 0.05). A predictive model constructed from multivariate analysis results stratified the monotherapy group (*n* = 69) into three subgroups, with scores of 0 (*n* = 5), 1 (*n* = 25), and 2 (*n* = 39), based on LDH (0 for < 265 and 1 for ≥ 265) and LMR (0 for ≥ 3.571 and 1 for < 3.571). Median PFS values were 0.57, 0.46, and 0.07 years for scores 0, 1, and 2, respectively (log‐rank test: *p* = 0.005 for score 0 vs. 2; *p* < 0.001 for score 1 vs. 2). The simple model combining LDH and LMR may predict PFS in patients with R/R aggressive ATL receiving mogamulizumab treatment. Since LDH and LMR are easily measurable in clinical practice, this model could help predict mogamulizumab efficacy and guide treatment decisions in this patient population.

**Trial Registration:** Registration number: UMIN000049135. Date of registration: October 17, 2022

## 1 Introduction

Adult T‐cell leukemia‐lymphoma (ATL), a rare and aggressive subtype of peripheral T‐cell lymphoma, is associated with poor prognosis [1]. The modified LSG15 (mLSG15) regimen (vincristine, cyclophosphamide, doxorubicin, and prednisolone; doxorubicin, ranimustine, and prednisolone; and vindesine, etoposide, carboplatin, and prednisolone [VCAP‐AMP‐VECP]) followed by allogeneic hematopoietic stem cell transplantation (allo‐HSCT) is a promising treatment option for aggressive ATL [2]. In patients ineligible for allo‐HSCT with CC chemokine receptor 4 (CCR4)‐positive aggressive ATL, two clinical studies have evaluated the sequential or concurrent use of cyclophosphamide, doxorubicin, vincristine, and prednisolone (CHOP) with mogamulizumab [3, 4]. Monotherapy with mogamulizumab, lenalidomide, tucidinostat, and valemetostat has been evaluated for the treatment of patients with relapsed/refractory (R/R) aggressive ATL in Japan [5, 6, 7, 8]. Despite treatment advances, patients with aggressive ATL generally have a poor prognosis [9], highlighting the need for improved treatment outcomes.

Mogamulizumab, an anti‐CCR4 monoclonal antibody, was approved in Japan for patients with CCR4‐positive aggressive ATL, as monotherapy for R/R ATL and in combination with chemotherapy for untreated cases [5, 6, 10]. While a treatment strategy based on clinical subclassification and prognostic factors has been suggested to improve ATL treatment outcomes with chemotherapy [11], classification methods for mogamulizumab treatment remain limited. Some patient factors associated with mogamulizumab treatment outcomes have been reported, including the development of skin rash after administration, mutations at the C‐terminal of the CCR4 gene, the percentage of Tax‐specific cytotoxic T lymphocyte (CTL) counts, soluble interleukin‐2 receptor (sIL‐2R) levels, serum lactate dehydrogenase (LDH) levels, and cluster of differentiation (CD)2^‐^CD19^+^ B‐cell counts [12, 13, 14, 15, 16, 17, 18]. Although the appearance of skin rash is recognized as a predictor of mogamulizumab efficacy, limited information is available to predict skin rash development before mogamulizumab administration. While CCR4 gene C‐terminal mutations, Tax‐specific CTL counts, and CD2^‐^CD19^+^ B‐cell counts are useful indices associated with treatment response, these factors are not routinely measured in clinical practices [13, 16, 18]. Furthermore, sIL‐2R and LDH are reported as prognostic factors [19, 20] rather than predictive indices for patients with ATL. Since immune‐related effects mediated by antibody‐dependent cellular cytotoxicity (ADCC) and regulatory T‐cell depletion are key mechanisms of action for mogamulizumab [21, 22], sIL‐2R or LDH alone does not directly reflect mogamulizumab treatment outcomes. Recent studies have shown that a combination of easily measurable clinical parameters representing immune and tumor status can predict the efficacy of monoclonal antibodies [23, 24]. This strategy is expected to be applicable to mogamulizumab treatment in patients with ATL.

In this analysis, we explored factors associated with the progression‐free survival (PFS) of mogamulizumab treatment in patients with CCR4‐positive aggressive ATL using data from clinical trials and proposed a simple model to predict mogamulizumab treatment outcomes based on routinely measured parameters.

## 2 Methods

### 2.1 Participants

This study is a pooled analysis using patient‐level data from three phase II clinical trials (NCT00920790 [5], NCT01173887 [10] and NCT01626664 [25]) and an observational clinical study (UMIN000013294 [26]). The detailed characteristics of the included studies are provided in Supporting Information S1.

Based on the content of patient consent forms from the clinical trials and study, the appropriateness of conducting this research was confirmed through an ethical review by the Research Ethical Review Committee of Kyowa Kirin Co., Ltd. (registration number: 2022_015). The institutional review boards of Kyoto University determined that an additional review of this research was not necessary because privacy information was protected by anonymization of all the data in each trial.

### 2.2 Procedure

For the analysis, patients were categorized into the following four groups (Supporting Information S2): the mogamulizumab monotherapy group, the mLSG15 and mogamulizumab combination treatment group, the investigator's choice (IC) group, and the mLSG15 group.

In this analysis, the observation period was defined as the time period between the earliest and latest recorded dates in a patient's medical records.

Factors measured in each trial were used for this analysis. Among the factors, lymphocyte count was calculated by subtracting actual abnormal lymphocyte count from actual lymphocyte count. If the result was 0 or less, it was set to 0. Monocyte count was derived from white blood cell (WBC) fraction and WBC count for the NCT00920790 and NCT01173887 trials. Neutrophil‐to‐lymphocyte count ratio (NLR), platelet‐to‐lymphocyte count ratio (PLR), and lymphocyte‐to‐monocyte count ratio (LMR) were calculated using the respective values.

### 2.3 Outcomes

The primary outcome of this analysis was to explore factors associated with PFS through univariate and multivariate analyses. Based on the factors identified in the multivariate analysis, a model was developed to categorize patient populations. Secondary outcomes included objective response rate (ORR), defined as the best response achieved (complete response [CR] + unconfirmed complete response [CRu] + partial response [PR]), as well as overall survival (OS) and the incidence of adverse events (AEs) related to skin and subcutaneous tissue in the population. The stratification results of PFS, ORR, and OS were investigated to evaluate the effects of the model on treatment outcomes. For both patients with R/R and chemotherapy‐naïve patients, PFS and OS were defined as in the original trials and study. If an allo‐HSCT was performed, the procedure was terminated as of the start date of the pretreatment. For OS analysis in the NCT01173887 and UMIN000013294 studies, patients were censored at the time of allo‐HSCT.

### 2.4 Statistical Analysis

For PFS and OS, survival curves were estimated using the Kaplan‐Meier method and compared between groups using the log‐rank test. Cox proportional hazards models were used to analyze PFS and OS by group. In the exploratory analysis of factors associated with PFS, variables with *p* < 0.1 in the univariate analysis were included in the multivariate analysis. As imputation of missing values was not conducted, patients with missing data were excluded from the analysis. Hazard ratios (HRs) and their 95% confidence intervals for the stratified population, as determined by the obtained model, were also estimated. Statistical significance was defined as a two‐tailed *p*‐value of < 0.05. The number and percentage of AEs were calculated for the overall population and by group using the stratification factor categories. As this was an exploratory study, no adjustments were made for multiple testing. All analyses were performed using the Statistical Analysis System (SAS) software, version 9.4 (SAS Institute, Cary, NC, USA).

## 3 Results

### 3.1 Patient Background and Baseline Data

In this analysis, 12 items routinely measured in clinical practice and three calculated indices—NLR, PLR, and LMR—were selected as variables (Table 1).

**TABLE 1:** Patient background and distribution of baseline values.

Data item	Differentiation	Mogamulizumab monotherapy group (*n* = 69)	Investigator's choice group (*n* = 24)	mLSG15 and mogamulizumab combination treatment group (*n* = 29)	mLSG15 group (*n* = 24)
Sex^a^	Male	33 (47.8)	10 (41.7)	12 (41.4)	16 (66.7)
	Female	36 (52.2)	14 (58.3)	17 (58.6)	8 (33.3)
Age (years)		60.0 (51.0–66.0)	50.5 (38.0–56.5)	61.0 (55.0–66.0)	63.5 (56.5–68.0)
Performance status^a^	0/1	47 (68.1)	17 (70.8)	26 (89.7)	22 (91.7)
	≥ 2	22 (31.9)	7 (29.2)	3 (10.3)	2 (8.3)
Corrected calcium value (mmol/L)		2.385 (2.270–2.495)	2.569 (2.320–2.897)	2.395 (2.320–2.470)	2.358 (2.258–2.557)
Albumin level (g/dL)		3.800 (3.400–4.100)	3.600 (3.100–4.160)	3.500 (3.300–3.900)	3.800 (3.150–4.150)
LDH level (IU/L)		422.0 (265.0–943.0)	510.0 (338.0–1424.0)	419.0 (320.0–512.0)	291.0 (210.5–537.5)
Ann arbor stage^a^	I/II	9 (13.0)	0 (0.0)	6 (20.7)	6 (25.0)
	III/IV	60 (87.0)	24 (100.0)	23 (79.3)	18 (75.0)
ATL subtype^a^	Acute type	33 (47.8)	12 (50.0)	20 (69.0)	17 (70.8)
	Lymphoma type	23 (33.3)	9 (37.5)	6 (20.7)	7 (29.2)
	Unfavorable chronic type	13 (18.8)	3 (12.5)	3 (10.3)	0 (0.0)
Monocyte count (× 10^9^/L)		0.574 (0.417–1.090)	0.754 (0.365–2.232)	0.664 (0.429–0.814)	0.526 (0.298–0.775)
Neutrophil count (× 10^9^/L)		3.655 (2.200–5.605)	2.905 (1.800–5.040)	4.330 (3.731–6.598)	4.042 (2.767–6.175)
Lymphocyte count (/μL)		1117 (754.0–2573)	1299 (810.0–5934)	1183 (709.0–1932)	1182 (767.0–1817)
Platelet count (× 10^9^/L)		201.5 (126.5–284.0)	189.0 (128.0–243.0)	213.0 (171.0–259.0)	208.5 (158.5–273.5)
Neutrophil‐to‐lymphocyte count ratio		2.427 (0.920–4.253)	1.393 (0.809–3.453)	3.333 (2.132–10.714)	3.400 (1.812–7.759)
Platelet‐to‐lymphocyte count ratio		146.1 (56.57–268.3)	130.5 (36.23–272.6)	205.3 (110.2–298.4)	170.1 (102.1–324.0)
Lymphocyte‐to‐monocyte count ratio		2.143 (1.200–3.571)	3.290 (1.098–5.429)	1.759 (0.750–2.833)	2.278 (1.234–5.860)

*Note:* All data are presented as median (Q1‐Q3), unless specified otherwise.

Abbreviations: ATL, adult T‐cell leukemia‐lymphoma; LDH, lactate dehydrogenase; mLSG15, modified LSG15; Q1, first quartile; Q3, third quartile.

^a^ Data are presented as *n* (%).

The mogamulizumab monotherapy group had a low lymphocyte count (median: 1117/μL). Neutrophil (median: 3655/μL), platelet (median: 201,500/μL), and monocyte (median: 574/μL) counts were within the normal range. NLR, PLR, and LMR were outside normal ranges due to low lymphocyte counts. Other groups showed similar trends.

With the exception of the IC group, which included only patients from outside Japan, the median age was ≥ 60 years. The proportion of patients with performance status ≥ 2 was higher in the monotherapy and IC groups. Median LDH values in all groups were > 290 IU/L.

### 3.2 Cox Proportional Hazards Model Analysis

**FIGURE 1:**
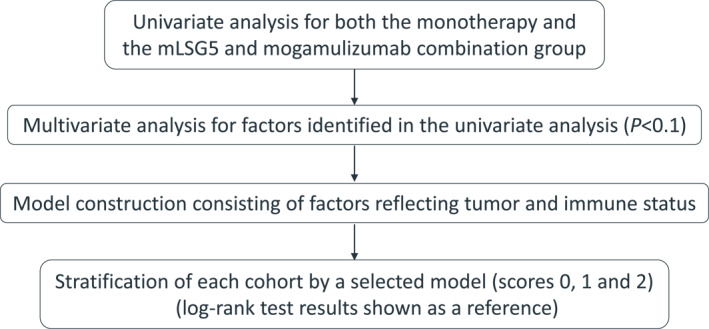
Analysis flow of this research. mLSG15, modified LSG15.

This exploratory analysis comprised the following: (1) univariate analysis, (2) multivariate analysis, (3) model construction based on tumor and immune status, and (4) stratification of each cohort using the model (Figure 1).

First, a univariate Cox proportional hazards model was applied using the 15 selected variables as explanatory variables and PFS as the outcome variable. Since long‐term survival with mogamulizumab has been reported in approximately 30% of patients, HRs were calculated for each factor using the 25th or 75th percentile as the cutoff for continuous variables.

The univariate analysis showed that age, albumin level, disease type, LDH, monocyte count, neutrophil count, and LMR were relevant factors in the monotherapy group (*p* < 0.1; Table 2). Among these, albumin level, LDH, disease type, monocyte count, neutrophil count, and LMR had *p*‐values < 0.05. Since this analysis aimed to explore the factors related to mogamulizumab efficacy, the mLSG15 group and IC groups were excluded from univariate and multivariate analyses. None of the explanatory variables were significant in the combination therapy group (data not shown).

**TABLE 2:** Factors associated with progression‐free survival in the mogamulizumab monotherapy group (univariate and multivariate analyses).

Data item	Category	No. of cases	Events	Per person‐year event occurrence rate (95% CI)	Univariate analysis		Multivariate analysis	
			Yes *n* (%)		HR (95% CI)	*p*‐value	HR (95% CI)	*p*‐value
Sex	Male^a^	33	24 (72.7)	3.44 (2.06, 4.81)	—	—	—	—
	Female	36	28 (77.8)	3.27 (2.06, 4.48)	1.01 (0.58–1.76)	0.972	—	—
Age (years)	≥ 66^a^	20	14 (70.0)	2.35 (1.12, 3.58)	—	—	—	—
	51–66	32	24 (75.0)	3.13 (1.88, 4.39)	1.22 (0.62–2.39)	0.569	1.67 (0.63–4.44)	0.302
	< 51	17	14 (82.4)	7.26 (3.46, 11.07)	1.99 (0.93–4.27)	0.078	3.51 (1.17–10.6)	0.025
Performance status	0/1^a^	47	37 (78.7)	2.85 (1.93, 3.77)	—	—	—	—
	≥ 2	22	15 (68.2)	5.80 (2.87, 8.74)	1.52 (0.81–2.86)	0.192	—	—
Corrected calcium value (mmol/L)	≥ 2.495^a^	17	13 (76.5)	5.51 (2.52, 8.51)	—	—	—	—
	2.270–2.495	33	24 (72.7)	2.44 (1.47, 3.42)	0.60 (0.30–1.21)	0.154	—	—
	< 2.270	15	12 (80.0)	3.76 (1.63, 5.89)	0.95 (0.42–2.12)	0.899	—	—
Albumin level (g/dL)	≥ 4.1	19	13 (68.4)	2.19 (1.00, 3.38)	0.39 (0.18–0.85)	0.019	0.22 (0.07–0.66)	0.007
	3.4–4.1	31	23 (74.2)	3.15 (1.86, 4.43)	0.51 (0.26–1.04)	0.063	0.61 (0.24–1.54)	0.295
	< 3.4^a^	15	13 (86.7)	6.15 (2.81, 9.49)	—	—	—	—
LDH level (IU/L)	≥ 943^a^	18	14 (77.8)	11.19 (5.33, 17.05)	—	—	—	—
	265–943	35	28 (80.0)	3.75 (2.36, 5.14)	0.44 (0.22–0.86)	0.017	0.46 (0.19–1.09)	0.078
	< 265	16	10 (62.5)	1.46 (0.56, 2.37)	0.18 (0.07–0.44)	< 0.001	0.21 (0.06–0.72)	0.012
Ann arbor stage	I/II^a^	9	6 (66.7)	3.32 (0.66, 5.98)	—	—	—	—
	III/IV	60	46 (76.7)	3.35 (2.38, 4.31)	0.99 (0.42–2.34)	0.978	—	—
ATL subtype	Acute type^a^	33	25 (75.8)	3.95 (2.40, 5.50)	—	—	—	—
	Lymphoma type	23	19 (82.6)	4.94 (2.72, 7.16)	1.08 (0.59–1.97)	0.806	0.85 (0.36–2.04)	0.722
	Unfavorable chronic type	13	8 (61.5)	1.49 (0.46, 2.52)	0.37 (0.16–0.87)	0.023	0.70 (0.24–2.02)	0.510
Monocyte count (× 10^9^/L)	> 1.090^a^	18	15 (83.3)	7.62 (3.76, 11.48)	—	—	—	—
	0.417–1.090	34	24 (70.6)	2.69 (1.61, 3.77)	0.40 (0.21–0.79)	0.008	1.68 (0.50–5.70)	0.404
	< 0.417	17	13 (76.5)	2.79 (1.27, 4.30)	0.52 (0.24–1.13)	0.097	2.96 (0.73–12.0)	0.129
Neutrophil count (× 10^9^/L)	≥ 5.605^a^	17	14 (82.4)	6.46 (3.07, 9.84)	—	—	—	—
	2.200–5.605	34	24 (70.6)	2.98 (1.79, 4.17)	0.47 (0.24–0.93)	0.030	0.48 (0.17–1.35)	0.167
	< 2.200	17	13 (76.5)	2.46 (1.12, 3.79)	0.43 (0.20–0.93)	0.033	0.37 (0.10–1.40)	0.142
Lymphocyte count (/μL)	≥ 2573	17	12 (70.6)	3.02 (1.31, 4.73)	1.01 (0.45–2.26)	0.983	—	—
	754–2573	34	26 (76.5)	4.05 (2.49, 5.61)	1.34 (0.68–2.63)	0.403	—	—
	< 754^a^	17	13 (76.5)	2.54 (1.16, 3.92)	—	—	—	—
Platelet count (× 10^9^/L)	≥ 284.0	17	15 (88.2)	5.16 (2.55, 7.77)	1.20 (0.55–2.60)	0.648	—	—
	126.5–284.0	34	24 (70.6)	2.90 (1.74, 4.06)	0.84 (0.41–1.71)	0.629	—	—
	< 126.5^a^	17	12 (70.6)	2.77 (1.20, 4.34)	—	—	—	—
Neutrophil‐to‐lymphocyte count ratio	≥ 4.253^a^	17	13 (76.5)	3.85 (1.76, 5.94)	—	—	—	—
	0.92–4.253	34	25 (73.5)	3.02 (1.84, 4.21)	0.84 (0.43–1.64)	0.602	—	—
	< 0.92	17	13 (76.5)	3.35 (1.53, 5.18)	0.77 (0.35–1.69)	0.514	—	—
Platelet‐to‐lymphocyte count ratio	≥ 268.284	17	14 (82.4)	5.73 (2.73, 8.73)	1.49 (0.67–3.31)	0.327	—	—
	56.572–268.284	34	25 (73.5)	2.72 (1.65, 3.78)	0.95 (0.47–1.94)	0.896	—	—
	< 56.572^a^	17	12 (70.6)	3.10 (1.34, 4.85)	—	—	—	—
Lymphocyte‐to‐monocyte count ratio	≥ 3.571	17	11 (64.7)	1.53 (0.63, 2.44)	0.38 (0.16–0.89)	0.026	0.40 (0.13–1.17)	0.093
	1.2–3.571	35	28 (80.0)	5.07 (3.19, 6.94)	1.11 (0.55–2.22)	0.768	1.42 (0.55–3.63)	0.468
	< 1.2^a^	15	12 (80.0)	4.32 (1.87, 6.76)	—	—	—	—

Abbreviations: ATL, adult T‐cell leukemia‐lymphoma; CI, confidence interval; HR, hazard ratio; LDH, lactate dehydrogenase.

^a^ Reference group for HR analysis.

The variance inflation factor calculation confirmed that there was no multicollinearity among the factors used in the multivariate analysis for the monotherapy group. In the multivariate analysis adjusted for age, albumin level, disease type, LDH, monocyte count, neutrophil count, and LMR, the factors age, albumin level, LDH, and LMR resulted in *p* < 0.1 (Table 2).

### 3.3 Scoring and Stratification of the Monotherapy Group

Based on the results of the multivariate analysis, two scoring models were developed using one factor (albumin level or LDH) related to clinical or tumor status and another factor (LMR) related to immune status. Model 1 used albumin level (scored as 0 for ≥ 4.1 and 1 for < 4.1) and LMR (scored as 0 for ≥ 3.571 and 1 for < 3.571), with total scores ranging from 0 to 2. Patients (*n* = 69) were stratified into three groups as follows: 7, 24, and 36 patients with total scores of 0, 1, and 2, respectively. Model 2 used LDH (scored as 0 for < 265 and 1 for ≥ 265) and LMR (scored as 0 for ≥ 3.571 and 1 for < 3.571), stratifying the monotherapy group into 5, 25, and 39 patients with total scores of 0, 1, and 2, respectively.

**FIGURE 2:**
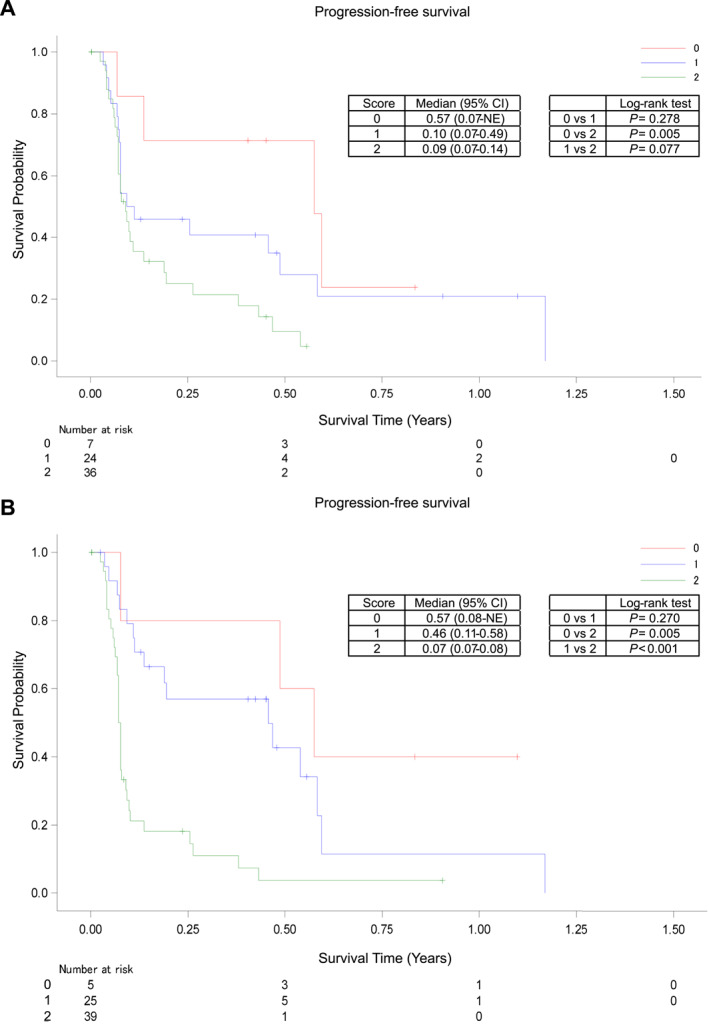
Summary of progression‐free survival for the mogamulizumab monotherapy group. (A) model 1^†^; (B) model 2^‡^. ^†^Albumin level (0 for ≥ 4.1 and 1 for < 4.1) and LMR (0 for ≥ 3.571 and 1 for < 3.571). ^‡^LDH (0 for < 265 and 1 for ≥ 265) and LMR (0 for ≥ 3.571 and 1 for < 3.571). CI, confidence interval; LDH, lactate dehydrogenase; LMR, lymphocyte‐to‐monocyte count ratio; NE, not estimable.

Stratification effects for PFS were analyzed for each score in models 1 and 2. The median PFS values in model 1 were 0.57, 0.10, and 0.09 years for scores 0, 1, and 2, respectively (log‐rank test: *p* = 0.005 for score 0 vs. 2; Figure 2A). In model 2, the median PFS values were 0.57, 0.46, and 0.07 years for scores 0, 1, and 2, respectively (log‐rank test: *p* = 0.005 for score 0 vs. 2; *p* < 0.001 for score 1 vs. 2; Figure 2B). The ORRs in model 1 were 71.4% (5/7 patients; 2 CR) for score 0, 45.8% (11/24 patients; 6 CR/CRu) for score 1, and 27.8% (10/36 patients; 2 CR/CRu) for score 2. The ORRs in model 2 were 60.0% (3/5 patients; 2 CR) for score 0, 64.0% (16/25 patients; 7 CR/CRu) for score 1, and 17.9% (7/39 patients; 1 CR) for score 2 (Table 3). While the median OS values in model 1 were 1.25 (score 0), 0.75 (score 1), and 0.43 (score 2) years, the median OS values in model 2 were not estimable (NE) for scores 0 and 1, and 0.32 years for score 2 (Supporting Information S3). Based on the results, model 2 was further investigated for safety and other predefined groups.

**TABLE 3:** ORRs of models 1 and 2 in the mogamulizumab monotherapy group.

	Model 1			Model 2		
	Score 0 (*n* = 7)	Score 1 (*n* = 24)	Score 2 (*n* = 36)	Score 0 (*n* = 5)	Score 1 (*n* = 25)	Score 2 (*n* = 39)
ORR^a^	5 (71.4)	11 (45.8)	10 (27.8)	3 (60.0)	16 (64.0)	7 (17.9)
CR/CRu^a^	2 (28.5)	6 (25.0)	2 (5.5)	2 (40.0)	7 (28.0)	1 (2.6)
PR^a^	3 (42.8)	5 (20.8)	8 (22.2)	1 (20.0)	9 (36.0)	6 (15.4)

Abbreviations: CR, complete response; CRu, unconfirmed complete response; ORR, objective response rate; PR, partial response.

^a^ Data are presented as *n* (%).

Regarding skin‐related AEs in model 2, all‐grade AEs occurred in all patients (*n* = 5) with score 0, 69.2% of patients (*n* = 18) with score 1, and in 39.5% of patients (*n* = 17) with score 2. Grade ≥ 3 AEs occurred in 2, 4, and 4 patients, respectively. One patient experiencing Stevens‐Johnson syndrome (grade ≥ 3) had a score of 0 (Table 4).

**TABLE 4:** Summary of skin‐related AEs (occurring in ≥ 10% of patients) in model 2.

Types of AEs	Score 0 *n* (%)		Score 1 *n* (%)		Score 2 *n* (%)	
	*n* = 5		*n* = 26		*n* = 43	
	All grade	Grade ≥ 3	All grade	Grade ≥ 3	All grade	Grade ≥ 3
Skin and subcutaneous tissue disorders^a^	5 (100.0)	2 (40.0)	18 (69.2)	4 (15.4)	17 (39.5)	4 (9.3)
Dermatitis^a^	1 (20.0)	0 (0.0)	1 (3.8)	0 (0.0)	0 (0.0)	0 (0.0)
Drug eruption^a^	2 (40.0)	0 (0.0)	5 (19.2)	0 (0.0)	2 (4.7)	0 (0.0)
Dry skin^a^	1 (20.0)	0 (0.0)	1 (3.8)	0 (0.0)	1 (2.3)	0 (0.0)
Eczema^a^	1 (20.0)	1 (20.0)	1 (3.8)	0 (0.0)	0 (0.0)	0 (0.0)
Pruritus^a^	1 (20.0)	0 (0.0)	2 (7.7)	1 (3.8)	5 (11.6)	0 (0.0)
Rash^a^	1 (20.0)	0 (0.0)	11 (42.3)	3 (11.5)	5 (11.6)	2 (4.7)
Skin hypopigmentation^a^	1 (20.0)	0 (0.0)	0 (0.0)	0 (0.0)	0 (0.0)	0 (0.0)
Stevens‐Johnson syndrome^a^	1 (20.0)	1 (20.0)	0 (0.0)	0 (0.0)	0 (0.0)	0 (0.0)

*Note:* Immune system disorders occurred in 2 patients (1 patient: grade ≥ 3) with score 1 and 2 patients (all grade) with score 2.

Abbreviation: AE, adverse event.

^a^ Data are presented as *n* (%).

For the IC group, the median PFS values in model 2 were NE, 0.22, and 0.07 years, while the median OS values were 2.07, 2.00, and 0.45 years for scores 0, 1, and 2, respectively. There were no clear differences across groups (Supporting Information S4). In the OS results, long‐term survival was observed after 0.5 years. Since the trial allowed crossover to mogamulizumab from IC, which may have influenced OS outcomes, the number of crossovers were analyzed. In model 2, there were 1, 8, and 9 crossovers for scores 0, 1, and 2, respectively (Supporting Information S5).

### 3.4 Model 2 Application to the Combination Treatment and mLSG15 Groups

Model 2 was applied to the combination treatment group (1, 9, and 19 patients with total scores of 0, 1, and 2, respectively). The median PFS was NE for score 0, 3.55 years for score 1, and 0.66 years for score 2. The median OS was NE for scores 0 and 1, and 1.25 years for score 2 (Supporting Information S6). ORRs were 100% (1/1 patient with PR) for score 0, 88.9% (8/9 patients; 7 CR/CRu) for score 1, and 84.2% (16/19 patients; 8 CR/CRu) for score 2.

In the mLSG15 group (5, 8, and 11 patients with total scores of 0, 1, and 2, respectively), although there were no significant differences for both PFS and OS, the median OS showed a stratification trend (NE, 1.65, and 0.95 years, respectively) (Supporting Information S7). ORRs were 60.0% (3/5 patients; 2 CR/CRu) for score 0, 62.5% (5/8 patients; 4 CR/CRu) for score 1, and 90.9% (10/11 patients; 2 CR/CRu) for score 2.

## 4 Discussion

This study aimed to propose a practical scoring system for mogamulizumab efficacy based on easily measurable parameters to guide treatment selection for patients with aggressive ATL. Since aggressive ATL has a poor prognosis, optimal and timely treatment is critical for patient survival. Mogamulizumab is efficacious in most patients with aggressive ATL due to high CCR4 expression on ATL cells [27, 28]. In addition to reported factors associated with mogamulizumab efficacy [29, 30], the practical scoring system is valuable. Our results suggest that the model consisting of LDH and LMR can predict the efficacy of mogamulizumab treatment in patients with R/R aggressive ATL.

The patients included in this analysis were aged 50–60 years, which is younger than a real‐world study [30]. The proportion of patients with PS ≥ 2 was higher in the monotherapy and IC groups compared with the mLSG15 and mogamulizumab combination treatment or mLSG15 groups, which were influenced by the trial being conducted outside of Japan and the inclusion of patients with R/R ATL. Median LDH values in all groups were elevated, indicating high tumor burdens in patients with ATL. We excluded the abnormal lymphocytes while calculating the lymphocyte count to minimize the influence of ATL cells on immune status because these cells are target tumor cells of mogamulizumab and reported to have regulatory T‐cell function [31]. Due to the low lymphocyte counts, the median value of LMR (2.143; first quartile [Q1]‐third quartile [Q3]: 1.200–3.571) in the monotherapy group was low compared with the median values in healthy subjects [32]. This result suggests that ADCC activity of mogamulizumab treatment might be weaker in patients with aggressive ATL than the healthy population.

The univariate analysis showed that age, albumin level, disease type, LDH, monocyte count, neutrophil count, and LMR were associated with PFS in the monotherapy group. Although age, albumin level, disease type, LDH, and monocyte count have been previously reported as factors associated with OS and/or event‐free survival for patients with aggressive ATL [12, 30, 33], neutrophil count and LMR were identified as significant explanatory factors for PFS in this analysis. However, in the combination group, none of the factors were found significant in the univariate analysis probably due to its small sample size and favorable PFS compared to the monotherapy group.

Based on the multivariate analysis, albumin level or LDH (clinical or tumor status) and LMR (immune status) were selected to develop models. Stratification of PFS by scores in models 1 (albumin and LMR) and 2 (LDH and LMR) showed that the prediction of efficacy with mogamulizumab monotherapy was higher in model 2 than in model 1. This result can be attributed to the fact that tumor‐related effects are reflected more directly by LDH than by albumin. Additionally, ORRs of scores 0 and 1 in model 2 were numerically higher compared with score 2, which suggests an association between mogamulizumab response and a combination of LDH and LMR. According to the incidence of skin‐related AEs by scores in model 2, a 29% difference in incidence rate between scores 1 and 2 at all grades was noted. Even when limiting to drug eruption and rash, patients with scores 0 and 1 experienced these events more frequently than those with score 2. These results imply a relationship between the scores in this model and skin‐related AEs because of an association between skin rash and mogamulizumab efficacy [34].

Although LDH has been reported as a prognostic factor for patients with ATL, the model cannot distinctly separate each population of the IC group based on PFS. This suggests that the model may be ineffective as a predictor of PFS following chemotherapy. Interestingly, a stratification trend was observed for OS in the IC group. Since the trial allowed crossover to mogamulizumab from IC after disease progression, each score of the OS results may have been influenced by the efficacy of mogamulizumab monotherapy after the crossover.

When model 2 was applied to the combination and mLSG15 groups, no clear differences were demonstrated between both groups. There was a trend of stratification for PFS and OS in the combination group, suggesting that this model may show the efficacy of mogamulizumab/mLSG15 combination therapy. However, due to almost the same ORRs across the scores and the small sample size, no clear conclusions were obtained.

There were several limitations to the present analyses. Due to the limited sample size in the shortened trials, the predictive model cannot be validated with the analyzed data. Additionally, the patients in the analyzed trials were selected based on stringent inclusion/exclusion criteria and did not take the latest treatment options. Therefore, further validation of the model will be necessary in a real‐world setting. Although the analysis included data from four different trials, no specific approach was used to address the inter‐trial heterogeneity. Also, as the data may contain unidentified confounders (e.g., differences in previous treatments), the integration is partially supportive of simulating real‐world settings.

The model can help predict both the efficacy and rash appearance of mogamulizumab treatment and can aid in treatment decision‐making for patients with aggressive ATL. Previous therapies are likely to influence LDH and LMR. However, because LDH and LMR can be monitored in clinical practice, these effects are reflected in the model. Patients with low LDH and/or high LMR are likely to have some time to prepare for the next treatment after mogamulizumab administration. However, other patients will need additional options sooner.

## 5 Conclusions and Future Directions

Using data from clinical trials in patients with aggressive ATL, we explored factors affecting mogamulizumab efficacy and proposed a simple model combining LDH and LMR to predict outcomes of mogamulizumab monotherapy for patients with R/R aggressive ATL. This practical scoring system appears to be useful for assessing treatment outcomes of mogamulizumab therapies. The applicability of this model to real‐world data will be investigated to validate its usefulness.

## Author Contributions

Y.S. contributed to research design, data analysis, data interpretation, and drafting and editing of the manuscript. K.M. and H.K. contributed to research design, data analysis, data interpretation, and manuscript preparation based on correct data interpretation. Y.N. contributed to research design, data collection, data analysis, data interpretation, and drafting of the manuscript and revising it critically for intellectual content. All authors approved the final version and agreed to be accountable for all aspects of the work.

## Ethics Statement

All studies included in this analysis were conducted in accordance with the Declaration of Helsinki, the International Conference on Harmonization consolidated Good Clinical Practice guidelines, and any applicable national and local laws and regulations. Written informed consent was obtained from all patients.

## Consent

An opt‐out method was used so that patients and their families could refuse the use of their data in the study.

## Conflicts of Interest

Yutaka Shimazu has no conflicts of interest to disclose. Kenta Murotani received grants from Kyowa Kirin Co., Ltd. during the conduct of the research. Hiroki Kitabayashi is an employee of Kyowa Kirin Co., Ltd. and owns stock in the company. Yukihiro Nishio is an employee of Kyowa Kirin Co., Ltd.

## Peer Review

The peer review history for this article is available at link https://www.webofscience.com/api/gateway/wos/peer-review/10.1002/hon.70114.

## Supporting information

Supporting Information S1

## Data Availability

The datasets generated during and/or analyzed during this study are available from the corresponding author upon reasonable request.
