# Supplementary material for: Exploratory Analysis of Practical Predictive Indices for the Efficacy of Mogamulizumab in Patients With Aggressive Adult T‐Cell Leukemia‐Lymphoma
Source: Hematol Oncol. 2025 Jun 28;43(4):e70114. doi: 10.1002/hon.70114 (PMC12205353; doi:10.1002/hon.70114)
Supplement: Supplementary file 1 — Supporting Information S1 [file HON-43-e70114-s001.docx]

**Title**

Exploratory analysis of practical predictive indices for the efficacy of mogamulizumab in patients with aggressive adult T-cell leukemia-lymphoma

**Authors:** Yutaka Shimazu^1,2^, Kenta Murotani^3^, Hiroki Kitabayashi^4^, Yukihiro Nishio^4^

**Affiliations:**

^1^Kyoto Innovation Center for Next Generation Clinical Trials and iPS Cell Therapy, Kyoto University Hospital, Kyoto, Japan

^2^Department of Early Clinical Development, Graduate School of Medicine, Kyoto University, Kyoto, Japan

^3^Biostatistics Center, Kurume University, Fukuoka, Japan

^4^Kyowa Kirin Co., Ltd., Tokyo, Japan

**Corresponding author:**

Yukihiro Nishio, PhD

Otemachi Financial City Grand Cube, 1-9-2 Otemachi, Chiyoda-ku, Tokyo 100-0004, Japan

Email: yukihiro.nishio.1u@kyowakirin.com

**Supporting Information S1** Characteristics of the included studies

| **Study registration number** | **Type of study** | **Study location** | **Study duration** | **Study objective** | **Patients included** | **Inclusion criteria** |
| --- | --- | --- | --- | --- | --- | --- |
| NCT00920790 [5] | Phase II | Japan | June 2009 to November 2010 | Efficacy and safety of mogamulizumab monotherapy in patients with relapsed CCR4-positive ATL | 27 | Patients aged ≥20 years with relapsed CCR4-positive aggressive ATL |
| NCT01626664 [25] | Phase II | Outside of Japan | June 2012 to February 2018 | Efficacy and safety of mogamulizumab as a single agent vs investigator’s choice in patients with R/R ATL | 71 | Patients aged ≥18 years with R/R aggressive ATL |
| NCT01173887 [10] | Phase II | Japan | July 2010 to April 2012 | Efficacy and safety of mLSG15 alone vs mogamulizumab and mLSG15 combination treatment in patients with chemotherapy-naïve CCR4-positive ATL | 53 | Patients aged ≥20 years and with newly diagnosed CCR4-positive aggressive ATL |
| UMIN000013294 [26] | Long-term, observational | Japan | October 2014 to March 2018 | Prognosis of patients with chemotherapy-naïve CCR4-positive ATL | 53 | Patients with chemotherapy-naïve CCR4‑positive ATL after the completion of NCT01173887 |

ATL, adult T-cell leukemia-lymphoma; CCR4, CC chemokine receptor 4; HTLV-1, human T-lymphotropic virus type 1; mLSG15, modified LSG15; R/R, relapsed/refractory.

**Supporting Information S2** Patient groups analyzed

| **Groups** | **Types of patients included** |
| --- | --- |
| The mogamulizumab monotherapy group | Patients with R/R CCR4-positive ATL who received mogamulizumab monotherapy in clinical trials were included |
| The mLSG15 and mogamulizumab combination treatment group | Patients with chemotherapy-naïve CCR4-positive ATL who received the mLSG15 and mogamulizumab combination treatment were included |
| The investigator’s choice group | Patients with R/R ATL who received the investigator's choice treatment were included |
| The mLSG15 group | Treatment-naïve patients who received mLSG15 were included |

Investigator’s choice: pralatrexate, GemOx, or DHAP.

ATL, adult T-cell leukemia-lymphoma; CCR4, CC chemokine receptor 4; DHAP, dexamethasone, high-dose cytarabine, and cisplatin; GemOx, gemcitabine and oxaliplatin; mLSG15, modified LSG15; R/R, relapsed/refractory.

**Supporting Information S3** Summary of overall survival for the mogamulizumab monotherapy group. (A) model 1^†^; and (B) model 2^‡^

B

A


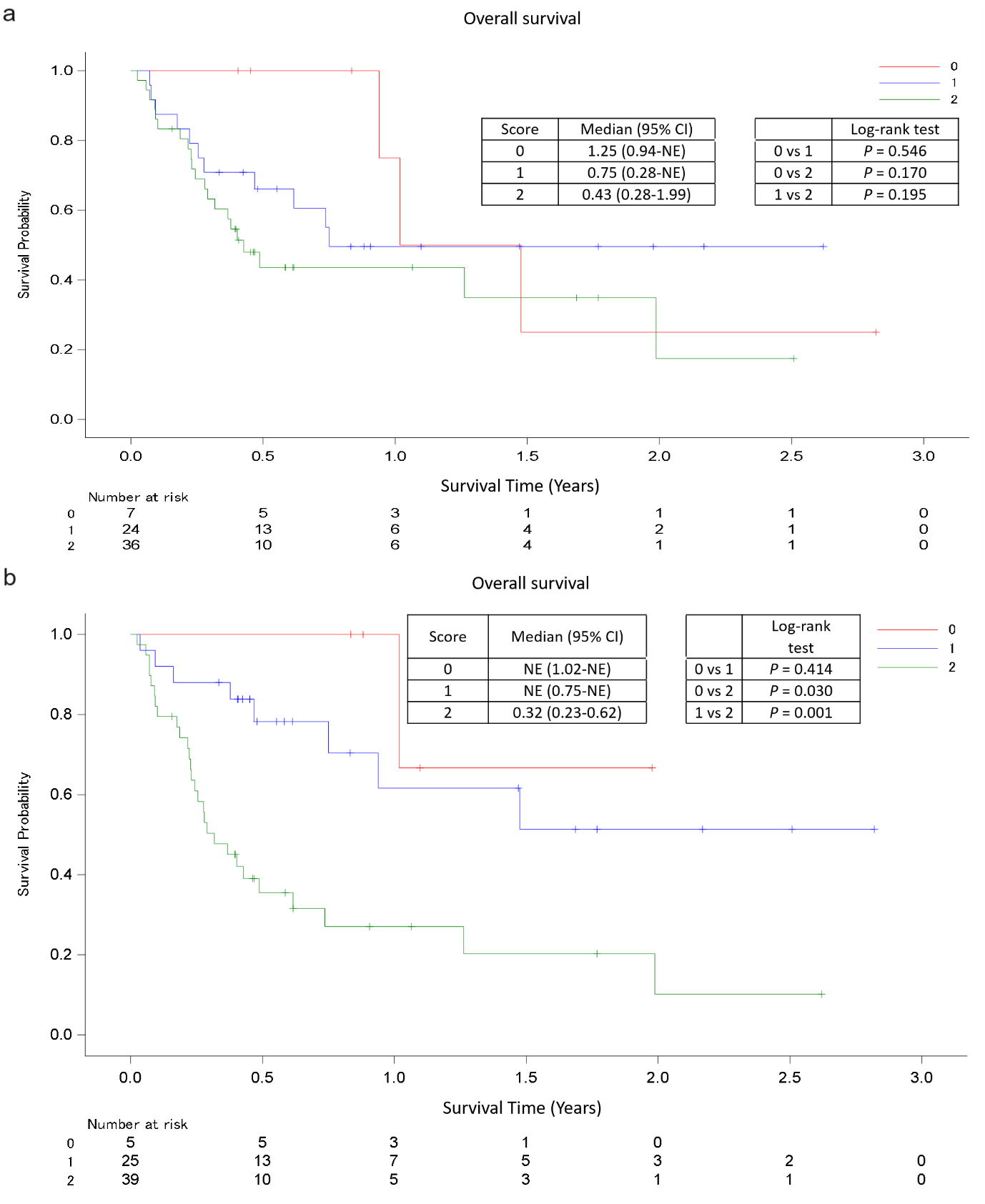


^†^Albumin level (0 for ≥4.1 and 1 for <4.1) and LMR (0 for ≥3.571 and 1 for <3.571).

^‡^LDH (0 for <265 and 1 for ≥265) and LMR (0 for ≥3.571 and 1 for <3.571).

CI, confidence interval; LDH, lactate dehydrogenase; LMR, lymphocyte-to-monocyte count ratio; NE, not estimable.

**Supporting Information S4** Summary of (A) progression-free survival and (B) overall survival for the investigator’s choice group (model 2^†^)

A

Progression-free survival


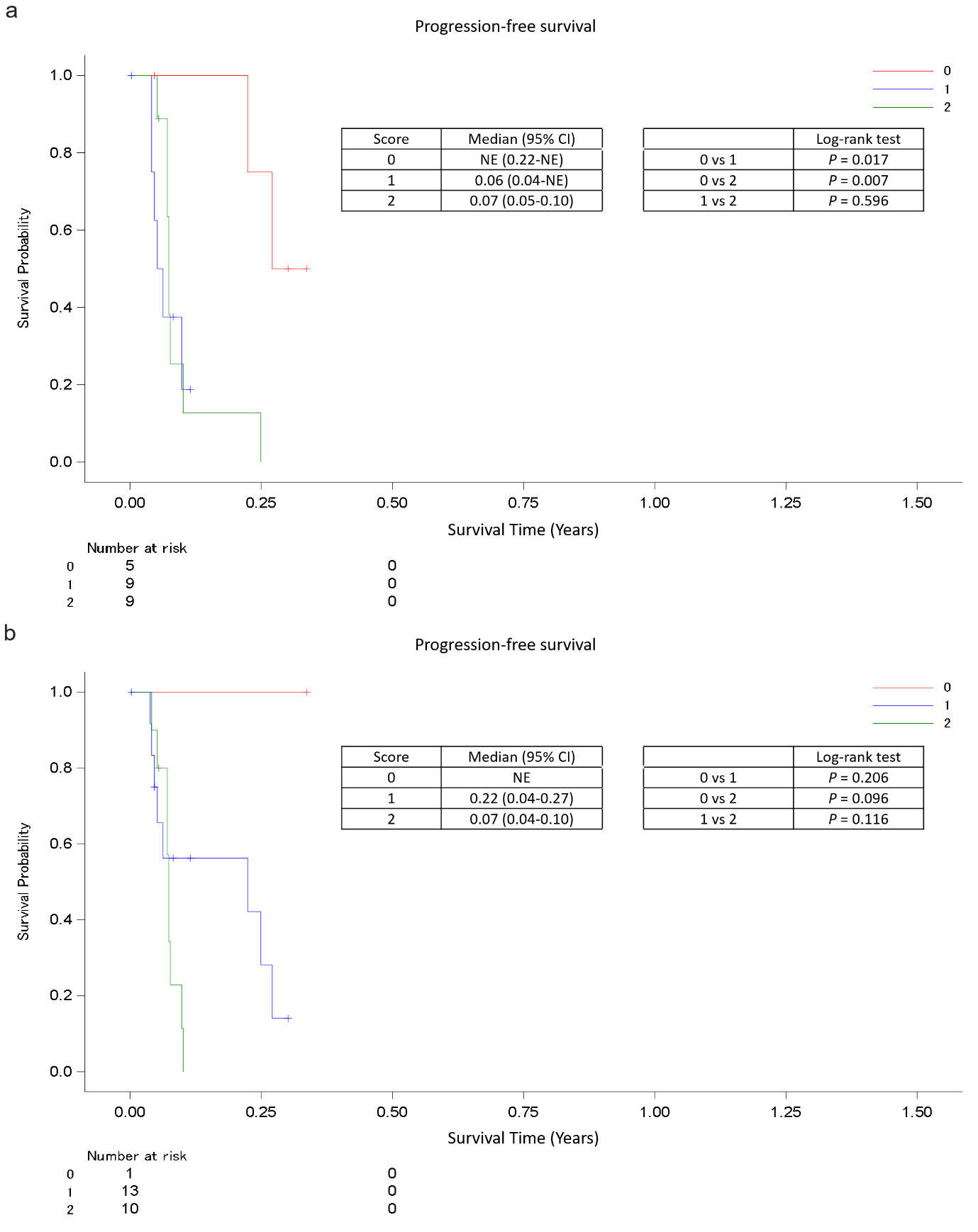

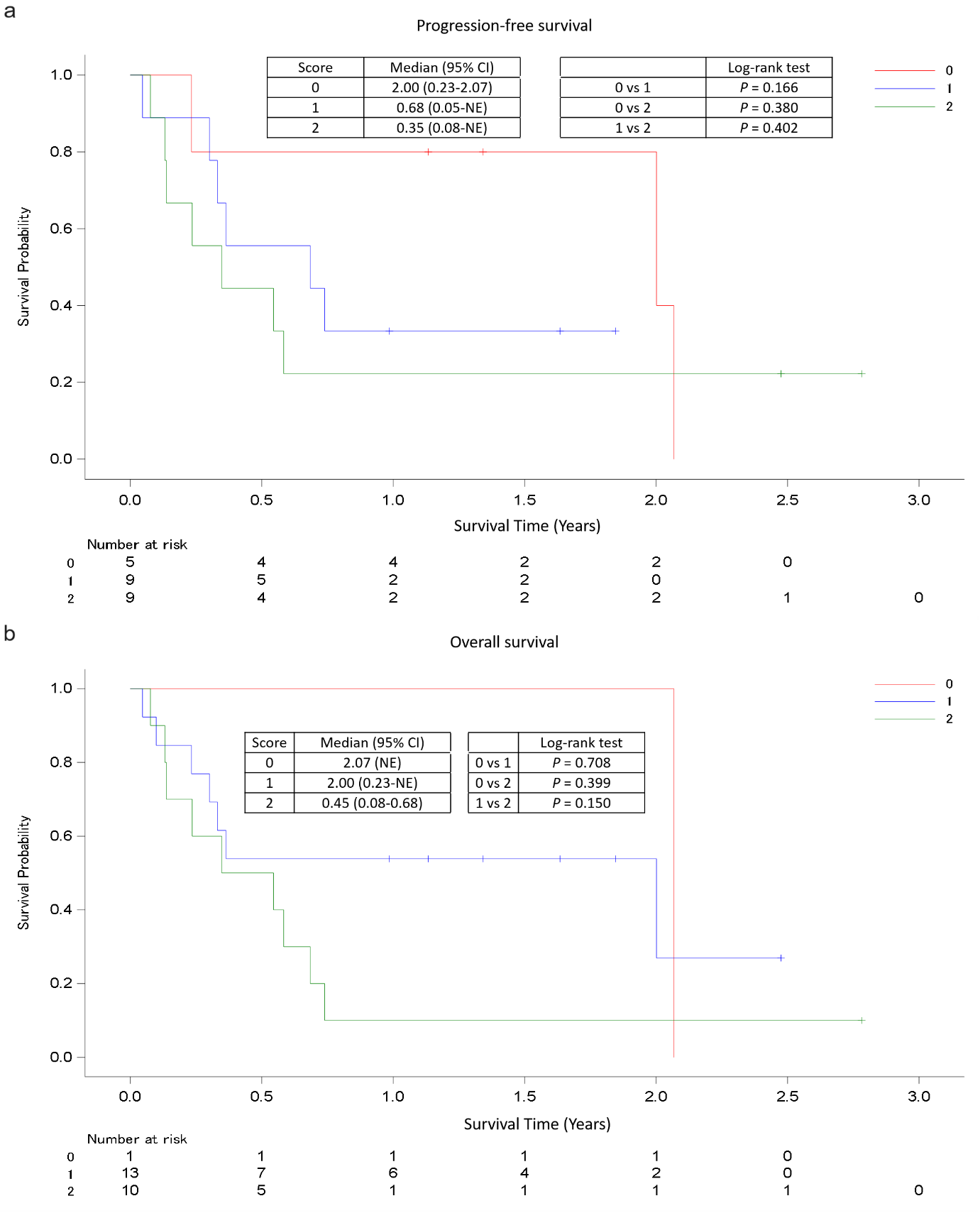


B

^†^LDH (0 for <265 and 1 for ≥265) and LMR (0 for ≥3.571 and 1 for <3.571).

CI, confidence interval; LDH, lactate dehydrogenase; LMR, lymphocyte-to-monocyte count ratio; NE, not estimable.

**Supporting Information S5** Numbers of patients receiving mogamulizumab and allo-HSCT after the crossover and the trial

|  | **Category** | **mLSG15 and mogamulizumab** | | **mLSG15** | | **Investigator’s choice** |
| --- | --- | --- | --- | --- | --- | --- |
|  |  | **Mogamulizumab** | **Allo-HSCT** | **Mogamulizumab** | **Allo-HSCT** | **Mogamulizumab** |
| Model 2^†^  LDH + LMR | Score 0 | 0 | 1 | 2 | 2 | 1 |
|  | Score 1 | 4 | 0 | 2 | 1 | 8 |
|  | Score 2 | 0 | 3 | 1 | 5 | 9 |

^†^LDH (0 for <265 and 1 for ≥265) and LMR (0 for ≥3.571 and 1 for <3.571).

Allo-HSCT, allogeneic hematopoietic stem cell transplantation; LDH, lactate dehydrogenase; LMR, lymphocyte-to-monocyte count ratio; mLSG15, modified LSG15.

**Supporting Information S6** Summary of (A) progression-free survival and (B) overall survival for the mLSG15 and mogamulizumab combination group (model 2^†^)


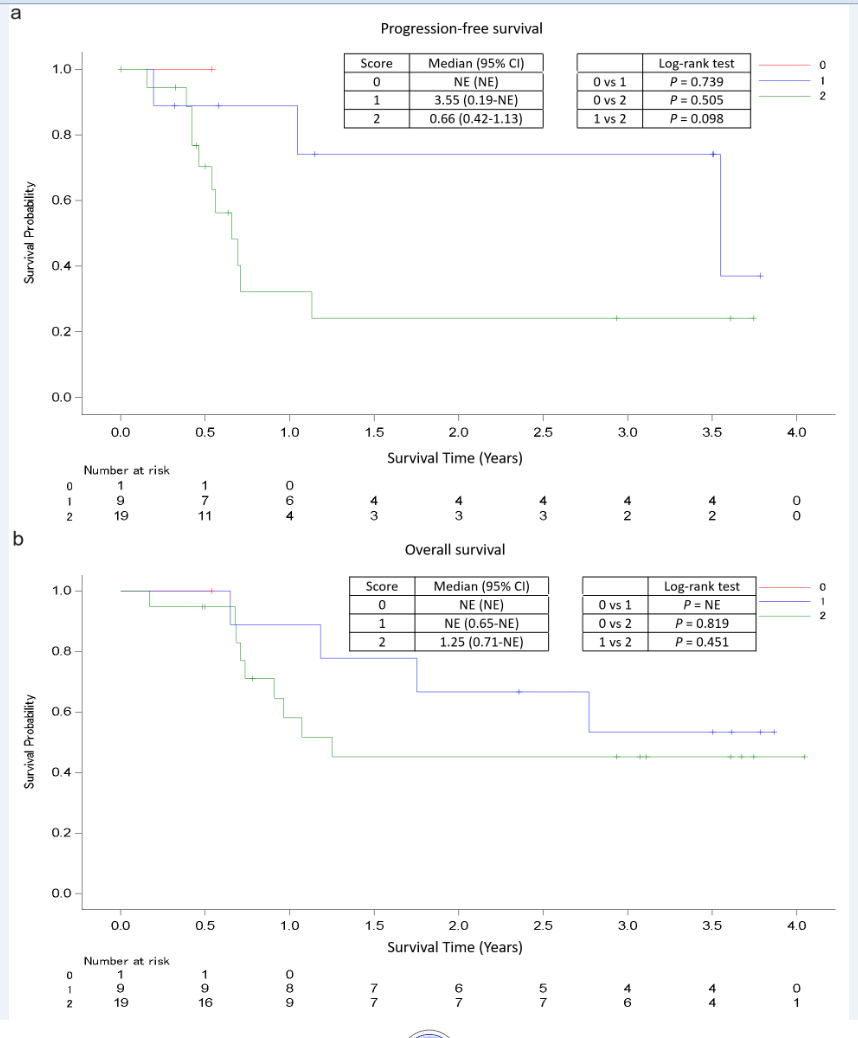


A

B

^†^LDH (0 for <265 and 1 for ≥265) and LMR (0 for ≥3.571 and 1 for <3.571).

CI, confidence interval; LDH, lactate dehydrogenase; LMR, lymphocyte-to-monocyte count ratio; mLSG15, modified LSG15; NE, not estimable.

**Supporting Information S7** Summary of (A) progression-free survival and (B) overall survival for the control (mLSG15) group (model 2^†^)


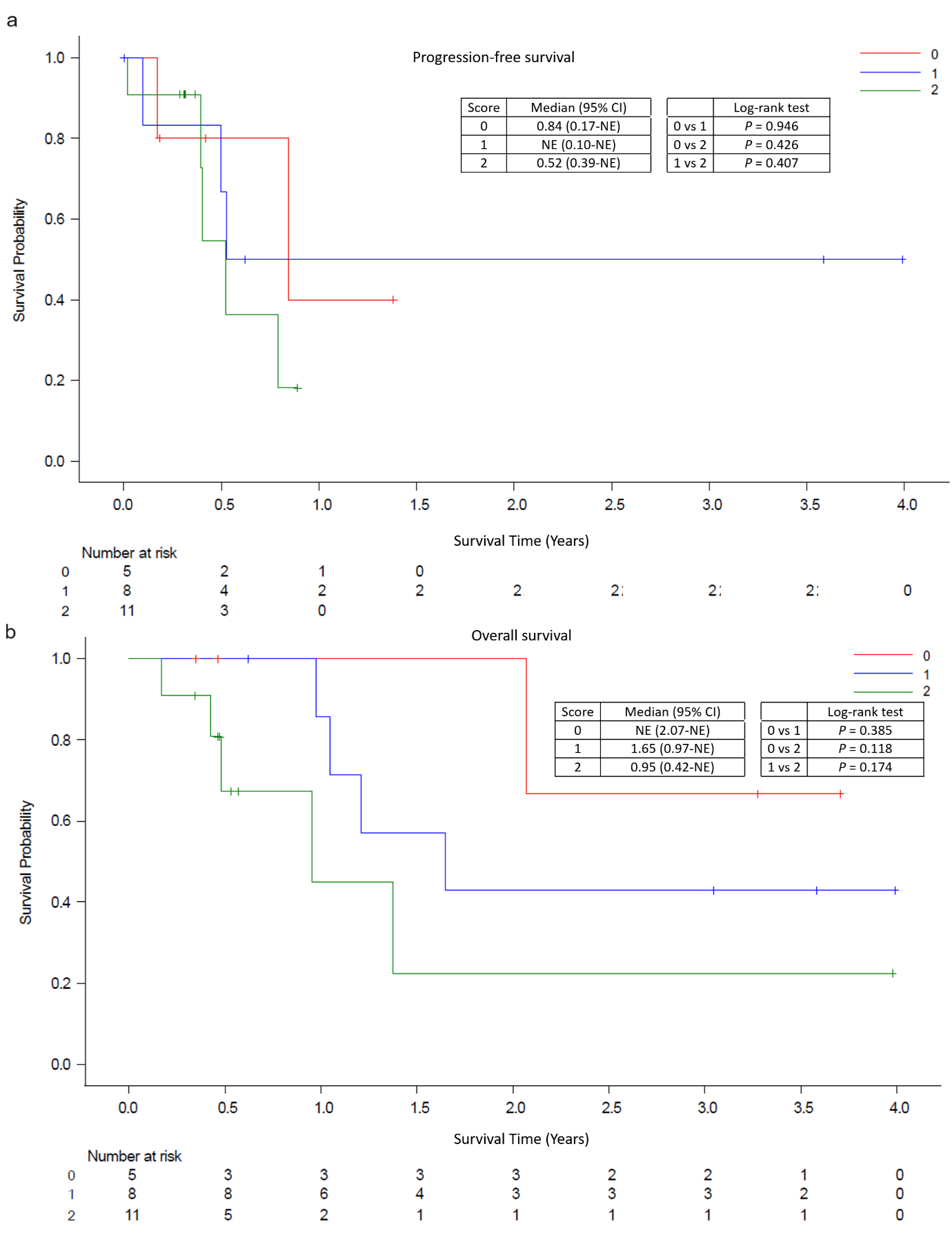


A

B

**LDH (0 for < 265 and 1 for ≥ 265) and LMR (0 for ≥ 3.571 and 1 for < 3.571)*

*LDH, lactate dehydrogenase; LMR, Lymphocyte-to-monocyte count ratio; mLSG15, Modified LSG15, NE, Not estimable*

^†^LDH (0 for <265 and 1 for ≥265) and LMR (0 for ≥3.571 and 1 for <3.571).

CI, confidence interval; LDH, lactate dehydrogenase; LMR, lymphocyte-to-monocyte count ratio; mLSG15, modified LSG15; NE, not estimable.
